# Patient compliance with touchdown weight bearing after microfracture treatment of talar osteochondral lesions

**DOI:** 10.1186/s13018-017-0548-5

**Published:** 2017-03-20

**Authors:** Gökhan Polat, Gökhan Karademir, Ekin Akalan, Mehmet Aşık, Mehmet Erdil

**Affiliations:** 1 Department of Orthopaedics and Traumatology, Istanbul University, Istanbul Medical Faculty, Çapa-Fatih, Istanbul 34093 Turkey; 2 Faculty of Science Health Physiotherapy & Rehab. Division, Istanbul University, Istanbul, Turkey; 3 Department of Orthopaedics and Traumatology, Istanbul Medipol University, Istanbul, Turkey

**Keywords:** Microfracture treatment, Patient compliance, Talar osteochondral lesion, Touchdown weight bearing

## Abstract

**Background:**

The aim of this study was to prospectively evaluate the compliance of our patients with a touchdown weight bearing (without supporting any weight on the affected side by only touching the plantar aspect of the foot to the ground to maintain balance to protect the affected side from mechanical loading) postoperative rehabilitation protocol after treatment of talar osteochondral lesion (TOL).

**Methods:**

Fourteen patients, who had been treated with arthroscopic debridement and microfracture, were followed prospectively. The patients were evaluated for weight bearing compliance with using a stationary gait analysis and feedback system at the postoperative first day, first week, third week, and sixth week.

**Results:**

The mean visual analog scale (VAS) scores of the patients at the preoperative, postoperative first day, first week, third week, and sixth weeks were 5.5, 5.9, 3.6, 0.9, and 0.4, respectively. The decrease in VAS scores were statistically significant (*p <* 0.0001). First postoperative day revealed a mean value of transmitted weight of 4.08% ±0.8 (one non-compliant patient). The mean value was 4.34% ±0.8 at the first postoperative week (two non-compliant patients), 6.95% ±2.3 at the third postoperative week (eight non-compliant patients), and 10.8% ±4.8 at the sixth postoperative week (11 non-compliant patients). In the analysis of data, we found a negative correlation between VAS scores and transmitted weight (Kendall’s tau *b* = −0.445 and *p =* 0.0228).

**Conclusions:**

Although patients were able to learn and adjust to the touchdown weight bearing gait protocol during the early postoperative period, most patients became non-compliant when their pain was relieved. To prevent this situation of non-compliance, patients should be warned to obey the weight bearing restrictions, and patients should be called for a follow-up at the third postoperative week.

## Background

Microfracture treatment is the most frequently performed bone marrow stimulation (BMS) technique for less than 1.5 cm^2^ full-thickness cartilage lesions and is accepted as the primary surgical procedure for talar osteochondral lesions (TOL) by many authors [1–9]. In addition to ensuring that the patient’s condition indicates microfracture treatment and using the proper surgical technique, postoperative rehabilitation involving non-weight bearing exercises for the affected area is crucial for the success of microfracture treatment [10–13].

Although there are some controversies regarding the postoperative rehabilitation of the TOL that were treated with microfracture, most of the surgeons allowed their patients with a non-weight bearing or touchdown weight bearing walking pattern in their practice. In addition, compliance to the rehabilitation protocol after surgery is an essential factor in the success of the treatment of TOL [14–17]. Touchdown weight bearing is defined as not supporting any weight on the affected side by only touching the plantar aspect of the foot to the ground to maintain balance to protect the affected side from mechanical loading. The aim of this study was to prospectively evaluate the compliance of our patients with a touchdown weight bearing postoperative rehabilitation protocol after treatment of TOL and compare their compliance to that of a control group of 10 healthy volunteers.

## Methods

Between March 2015 and November 2015, 14 patients who had undergone arthroscopic debridement and microfracture treatment for TOL were prospectively evaluated for this study. Patients (between 17 and 65 years) with TOL lesions that were smaller than 1.5 cm^2^ according to the magnetic resonance imaging (MRI) measurements and had no subchondral cysts, were included in this study. We recorded the demographics of the patients and their education level.

Patients were prepared in the supine position with a tourniquet on the extremity being operated on. Standard anteromedial and anterolateral portals were used with non-invasive distraction for ankle arthroscopy. After debridement and curettage of the lesion, a viable subchondral bone was obtained. Three- to four-millimeter-spaced holes were created via microfracture according to lesion size. The surgery ended with tourniquet release, and fat droplets and blood outflow were observed in the microfracture holes.

A standard microfracture postoperative rehabilitation protocol was applied for all patients. The patients were allowed to walk using two crutches with touchdown weight bearing. Full weight bearing was allowed 6 weeks postoperatively, at which time strengthening exercises were initiated.

**Fig. 1:**
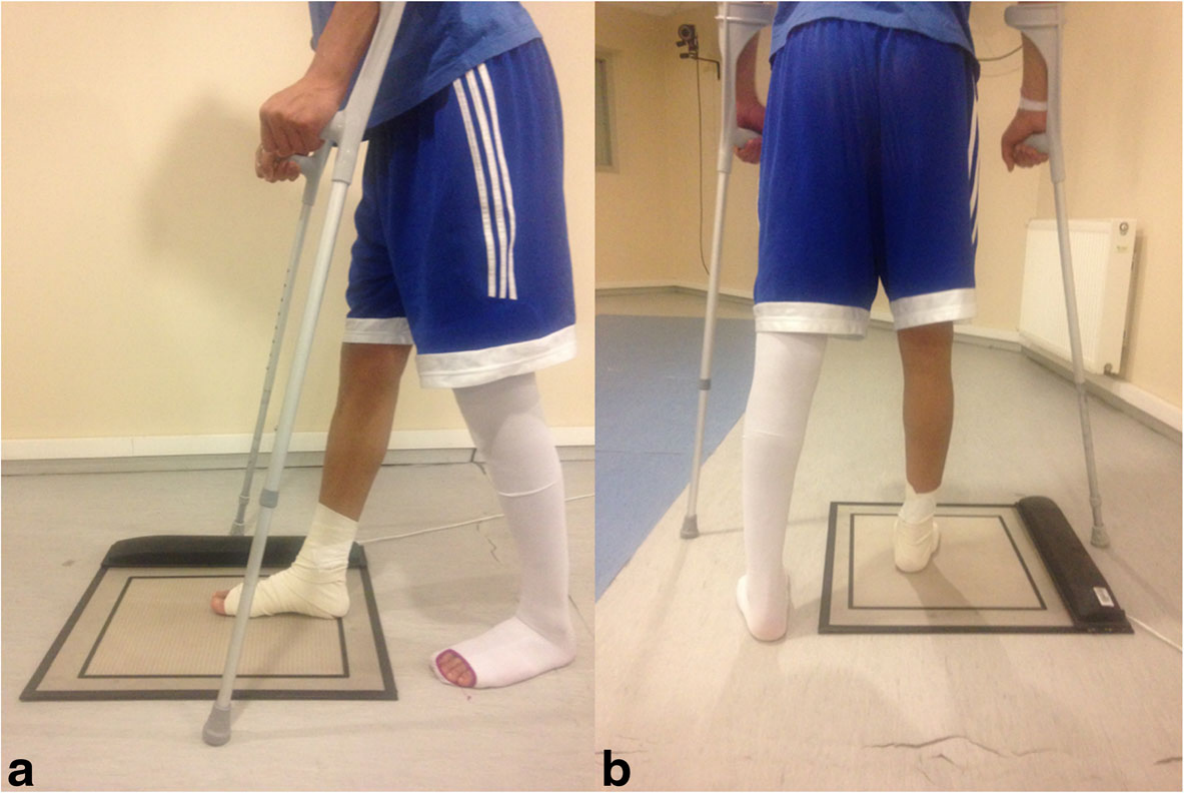
**a**, **b** Clinical pictures of a patient (number 6, 21-year-old male) during the analysis of weight bearing at the gait analysis laboratory

In the postoperative rehabilitation program, all patients were mobilized on the first postoperative day with touchdown weight bearing using two crutches. Before mobilization, the method of touchdown weight bearing for the operated extremity was shown to the patients by two surgeons. All patients walked on the platform for six cycles for four different times during postoperative follow-up. The maximum foot reaction-force during the gait cycle is measured as an absolute value, and this value is converted into a percentage according to the patient’s body weight. All patients walked on the platform for six cycles for one analysis and the mean values of these had taken under review (Fig. 1).

Because of the uncertainty of normal limits of this walking pattern in the literature, to determine the limit for weight transmission with touchdown weight bearing, a group of 10 healthy individuals were tested. The healthy individuals were only informed about the basic setup and were asked to perform touchdown weight bearing similar to the TOL patients. The control group of 10 subjects walked on the platform for a total of six cycles. Based on the results of the healthy individuals, a mean basal limit was determined for the percentage of weight transmission to the ground during touchdown weight bearing walking.

The patients were evaluated for weight bearing using a Medscan System (Tekscan®, Inc. Boston, USA), a stationary gait analysis and feedback system at the gait analysis laboratory of our clinic. The maximum foot reaction-force during the gait cycle is measured as an absolute value, and this value is converted into a percentage according to the patient’s body weight.

The data of the patients were recorded as percentage values at the first day, first week, third week, and sixth week postoperatively. No feedback about the test results was given to the patients, and the patients were asked to walk using the same pattern that they were shown after surgery at all evaluations. The patients were also evaluated for pain preoperatively, at the first day, first week, third week, and sixth week using the visual analog scale (VAS). In addition, the patients’ American Orthopedic Foot and Ankle Society (AOFAS) scores were determined as a functional assessment preoperatively and at the 12th week postoperatively.

This study was approved by the authors’ institutional review board, and all patients gave informed consent to participate in this study. Detailed information regarding the surgical interventions was provided to all patients. All patients signed an informed consent form that thoroughly explained the operative technique that they would undergo. The rehabilitation program was also explained to the patients.

Medcalc 15.11 for Mac® was used for all statistical analyses. The standard deviation and the mean value of the weight exerted on the leg were calculated for both groups. The paired sample *t*-test was used to compare the means of the two populations to determine the variables that were correlated. The Kendall’s tau correlation test was used for the correlation analysis of the weight exerted on the leg and the VAS and AOFAS scores because the sample size was small. The level of significance for all statistical tests was set at *p* < 0.05.

## Results

Ten male patients and four female patients composed our study group, and the mean age of the patients was 26.6 ± 6 years (range, 17–42 years). According to the Berndt and Harty classification, nine patients had type 3 lesions, and five patients had type 4 lesions [18]. Six patients had a college degree, and eight patients had a high school degree. There were no significant differences between compliance of the patients and age or academic degree of patients (n.s.).

In the healthy individuals group, the mean body weight percentage transmitted to the ground was 4.7 ± 1.2% (range, 3.2–5.4) and was used as the cut-off value for the limit of load for this walking pattern. Values above this limit were considered to indicate non-compliance with touchdown weight bearing.

The analysis of the patient data for the first postoperative day revealed a mean value of transmitted weight of 4.08% ±0.8. The mean value was 4.34% ±0.8 at the first postoperative week, 6.95% ±2.3 at the third postoperative week, and 10.8% ±4.8 at the sixth postoperative week. The mean values of the weight bearing analysis are summarized in Table 1.

**Table 1:** Demographics, the mean weight bearing values, VAS, and AOFAS scores of the patients are seen

	Values		Statistics
Mean age	26.6 ± 6 years (range 17–42)		
Male/female	10 male/4 female		
Education level	6 patients—college degree 8 patients—high school degree		
Mean level of weight bearing of control Subjects (*n =* 10):	4.7 ± 1.2% (range 3.2–5.4)		
Mean level of weight bearing of patients (*n =* 14)	Postoperative first day	4.08% ±0.8	Kendall’s tau *b =* −0.445 *p =* 0.0228
	Postoperative first week	4.34% ±0.8	
	Postoperative third weeks	6.95% ±2.3	
	Postoperative sixth week	10.8% ±4.8	
VAS scores	Preoperative	5.5	(*p <* 0.0001)
	Postoperative first day	5.9	
	Postoperative first week	3.6	
	Postoperative third weeks	0.9	
	Postoperative sixth week	0.4	
AOFAS score (preoperative)	75 ± 4.7 (range 68–82)		
AOFAS score (postoperative third months)	96.2 ± 5.4 (range 87–100)		(*p <* 0.0001)
Non-compliance of the patients during follow-up	Postoperative first day	1/14 (7.1%)	
	Postoperative first week	2/14 (14.3%)	
	Postoperative third weeks	8/14 (57.1%)	
	Postoperative sixth week	11/14 (78.5%)	

Functional status of the patients were evaluated by the AOFAS score, and the mean AOFAS score was 75 ± 4.7 (range, 68–82) preoperatively and 96.2 ± 5.4 (range, 87–100) postoperatively. Statistically significant improvements in AOFAS score were achieved (*p <* 0.001).

**Fig. 2:**
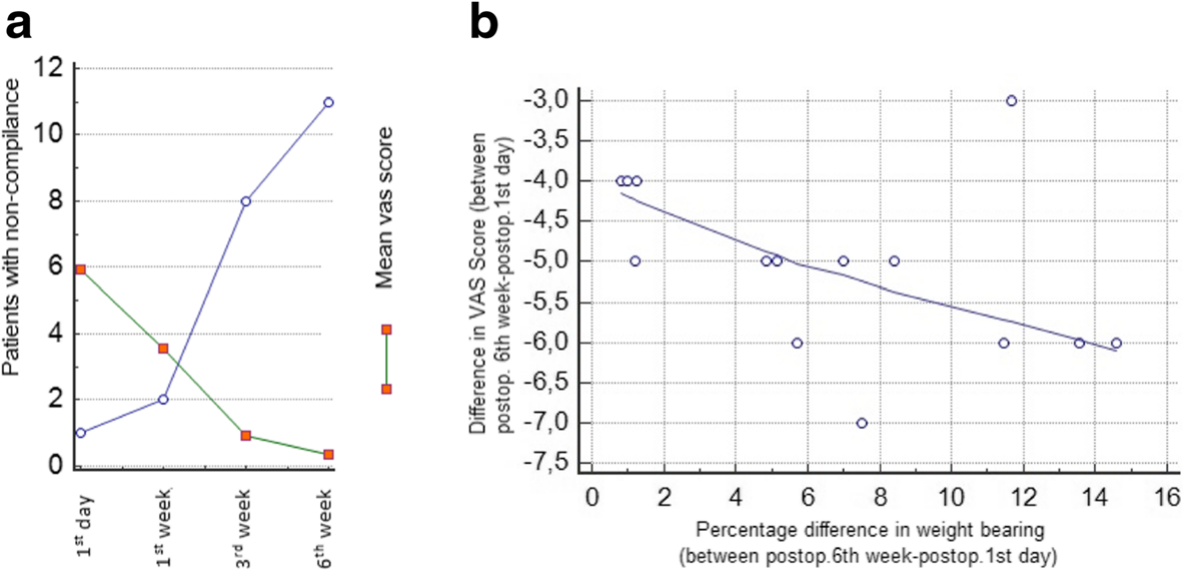
**a** Number of patients who were non-compliant with touchdown weight bearing, **b** Scatter diagram shows the correlation between the VAS score differences in the first and last control, and the difference of weight bearing values between first and last tests

The mean VAS scores of the patients on the preoperative, first postoperative day, and at the first, third, and sixth postoperative weeks were 5.5, 5.9, 3.6, 0.9, and 0.4, respectively. The decrease in VAS scores were statistically significant (*p <* 0.001). The transmitted weights of the patients and VAS scores were analyzed with Kendall’s tau correlation test, and we found a negative correlation between VAS score and transmitted weight (Kendall’s tau *b =* −0.445 and *p =* 0.0228) (Fig. 2a, b).

## Discussion

The microfracture technique is still the most frequently performed treatment method for full thickness cartilage lesions [5, 6]. Although there are controversies regarding the best postoperative rehabilitation program for microfracture patients, most authors prefer the implementation of a non-weight or touchdown weight bearing postoperative period to allow for the formation and maturation of the hyaline-like fibrocartilage tissue at the defect site [7–9, 15]. There are few publications on this issue, and some authors reported confusion regarding the use of weight bearing rehabilitation methods and issues with providing sufficient information to patients to ensure compliance with postoperative rehabilitation protocols [16, 17]. The purpose of the current study was to evaluate patient compliance with touchdown weight bearing walking throughout the postoperative period and to determine factors that may contribute to non-compliance with this rehabilitation program. The most important finding of this study is that although patients can learn and adjust to the touchdown weight bearing in the early postoperative period, most patients became non-compliant when their pain is relieved.

The proposed gait pattern after microfracture treatment is non-weight bearing, touchdown weight bearing, partial weight bearing, tolerated weight bearing, or full-weight bearing, according to the surgical procedure. In the literature, touchdown weight bearing was explained in details [19]. However, there is little information in the literature regarding transmitted weight to the affected side with this walking pattern, and the typical value of transmitted weight with ideal walking is unknown [17, 20]. To determine the limit for the amount of weight transmission to the affected side, we evaluated 10 healthy individuals as control group. The mean percentage of weight that was transmitted to the ground in the control group was 4.7 ± 1.2% (range, 3.2–5.4%). Therefore, we considered the upper limit of acceptable weight transmitted to the ground to be 4.7%, and we considered values above this cut-off value to indicate non-compliance.

In another study, Ruiz et al. analyzed 18 lower extremity trauma patients’ (acetabulum, tibia, femur, or ankle) compliance with the walking pattern of touchdown weight bearing [17]. In that study, the authors used a touchdown weight bearing limit of 25 lbs (11.33 kg) ± 10 lbs for all patients without giving a reference or a control measurement for this issue. At discharge, the average minimum and maximum weight bearing values were 3.2 and 30.2 lbs, respectively. Only 31% of steps were within the acceptable range of 15 to 35 lbs. At the first follow-up, the average minimum and maximum weight bearing values were 12.2 and 50.8 lbs, respectively. The authors reported that only 27% of steps were within the acceptable range. The authors evaluated both the amount of weight bearing and the percentage of steps within the acceptable range and reported that the majority of steps had less than the prescribed amount of weight bearing at discharge, whereas the majority of steps had more than the prescribed amount of weight bearing at the first follow-up. In that study the weight limit for touchdown weight bearing was 25 lbs; however, the appropriate limit has not been described in the literature. Due to the lack of a standard weight bearing limit for touchdown weight bearing, in our study we determined the limit for transmitted weight using a group of 10 healthy individuals. We measured the weight transmitted to the ground in these individuals and obtained a percentage value by dividing this transmitted weight by the body weight to standardize the values for all healthy individuals. The mean value of transmitted weight was 4.7 ± 1.2% (range, 3.2–5.4) and was used as the cut-off value for non-compliance. Using this cut-off value, 1 patient (7.1%), 2 patients (14.2%), 8 patients (56.8%), and 11 patients (78%) were non-compliant at the first day, first week, third week, and sixth week postoperatively.

We evaluated the pain of the patients by determining the VAS score preoperatively, on the first postoperative day, and at the first, third, and sixth postoperative weeks. We observed a significant decrease in the VAS scores of the patients during the postoperative period. We found a negative correlation between the VAS score and patient non-compliance with touchdown weight bearing (Kendall’s tau rank correlation coefficient *b =* −0.445 and *p =* 0.0228). Based on these results, we reject the null hypothesis of mutual independence between the VAS score and touchdown weight bearing rankings. Furthermore, the *p* value of 0.0228 indicates that the detected negative correlation is not coincidental with 95% confidence.

Compliance with weight bearing restrictions is closely related to good clinical outcomes of patients following some lower extremity surgeries, and they are usually prescribed to patients by the surgeons. However, there are some controversies regarding weight bearing restrictions, such as the definitions of weight bearing patterns and the best way to teach such patterns to patients [16]. There are some studies in the literature related to this issue, and they reported that patients are not able to walk in this limited weight bearing walking pattern [16, 21]. In our study, we found that the patients learned, adapted, and obeyed the weight restrictions in the first postoperative week. However, this compliance did not continue throughout the postoperative period, especially after 3 weeks. We believe that this decrement of compliance may be related to the decrement of pain and the psychological desire of the patient to test his/her operated leg. Our study results support a relationship between pain and non-compliance with weight bearing recommendations, and we found a strong negative correlation between the VAS score and the transmitted weight of the patients.

The main limitation of this study was the small sample size. However, the study group was homogenous and received a standard treatment, and no patients were lost during the follow-up. Another limitation of our study is the lack of a reference for the ideal amount of weight transmission to the ground with touchdown weight bearing. Additionally, we investigated patient compliance with weight bearing following one surgical procedure. Another limitation of this study is the lack of information regarding patients’ behaviors on non-testing days. We were not able to continuously evaluate the patients’ compliance with the touchdown weight bearing gait protocol.

## Conclusions

Postoperative rehabilitation after arthroscopic treatment of TOL is an important factor that affects the quality and endurance of the regenerated cartilage. Although patients were able to learn and adjust to the touchdown weight bearing gait protocol during the early postoperative period, most patients became non-compliant when their pain was relieved. To prevent this situation of non-compliance, patients should be warned to obey the weight bearing restrictions, and patients should be called for a follow-up at the third postoperative week.

## References

[1] Goldberg VM, Caplan AI (1999). Biologic restoration of articular surfaces. AAOS Instr Course Lect.

[2] Steadman JR, Rodkey WG, Rodrigo JJ. Microfracture: Surgical technique and rehabilitation to treat chondral defects. Clin Orthop Relat Res. 2001;(391 Suppl):S362-9. Review. doi:10.1016/S1048-6666(01)80019-7. 10.1097/00003086-200110001-00033 11603719

[3] Becher C, Driessen A, Thermann H (2008). Microfracture technique for the treatment of articular cartilage lesions of the talus. Orthopade.

[4] Li S, Li H, Liu Y, Qu F, Wang J, Liu C (2014). Clinical outcomes of early weight-bearing after arthroscopic microfracture during the treatment of osteochondral lesions of the talus. Chin Med J (Engl).

[5] Farr J, Cole B, Dhawan A, Kercher J, Sherman S (2011). Clinical cartilage restoration. Evolution and overview. Clin Orthop Relat Res.

[6] Clanton TO, Johnson NS, Matheny LM (2014). Outcomes following microfracture in grade 3 and 4 articular cartilage lesions of the ankle. Foot Ankle Int.

[7] Zengerink M, Struijs PA, Tol JL, van Dijk CN (2010). Treatment of osteochondral lesions of the talus: a systematic review. Knee Surg Sports Traumatol Arthrosc.

[8] Van Bergen CJ, Kox LS, Maas M, Sierevelt IN, Kerkhoffs GM, van Dijk CN (2013). Arthroscopic treatment of osteochondral defects of the talus: outcomes at eight to twenty years of follow-up. J Bone Joint Surg Am.

[9] Savage-Elliott I, Ross KA, Smyth NA, Murawski CD, Kennedy JG (2014). Osteochondral lesions of the talus: a current concepts review and evidence-based treatment paradigm. Foot Ankle Spec.

[10] Hurst JM, Steadman JR, O’Brien L, Rodkey WG, Briggs KK (2010). Rehabilitation following microfracture for chondral injury in the knee. Clin Sports Med.

[11] Reinold MM, Wilk KE, Macrina LC, Dugas JR, Cain EL (2006). Current concepts in the rehabilitation following articular cartilage repair procedures in the knee. J Orthop Sports Phys Ther.

[12] Van Eekeren IC, Reilingh ML, van Dijk CN (2012). Rehabilitation and return-to-sports activity after debridement and bone marrow stimulation of osteochondral talar defects. Sports Med.

[13] Choi WJ, Jo J, Lee JW (2013). Osteochondral lesion of the talus: prognostic factors affecting the clinical outcome after arthroscopic marrow stimulation technique. Foot Ankle Clin.

[14] Assche DV, Caspel DV, Staes F, Saris DB, Bellemans J, Vanlauwe J, Luyten FP (2011). Implementing one standardized rehabilitation protocol following autologous chondrocyte implantation or microfracture in the knee results in comparable physical therapy management. Physiother Theory Pract.

[15] Lee DH, Lee KB, Jung ST, Seon JK, Kim MS, Sung IH (2012). Comparison of early versus delayed weightbearing outcomes after microfracture for small to midsized osteochondral lesions of the talus. Am J Sports Med.

[16] Rubin G, Monder O, Zohar R, Oster A, Konra O, Rozen N (2010). Toe-touch weight bearing: myth or reality?. Orthopedics.

[17] Ruiz FK, Fu MC, Bohl DD, Hustedt JW, Baumgaertner MR, Leslie MP, Grauer JN (2014). Patient compliance with postoperative lower extremity touch-down weight-bearing orders at a level I academic trauma center. Orthopedics.

[18] Berndt AL, Harty M. Transchondral fractures (osteochondritis dissecans) of the talus. J Bone Joint Surg Am. 1959;41:988–1020. http://journals.lww.com/jbjsjournal/Abstract/1959/41060/Transchondral_Fractures__Osteochondritis.2.aspx. 13849029

[19] Pierson, F. Principles and Techniques of Patient Care, Third Edition. Philadelphia: WB Saunders Company; 2002. p.208.

[20] Haller JM, Potter MQ, Kubiak EN (2013). Weight bearing after a periarticular fracture: what is the evidence?. Orthop Clin North Am.

[21] Tveit M, Kärrholm J (2001). Low effectiveness of prescribed partial weight bearing. Continuous recording of vertical loads using a new pressure-sensitive insole. J Rehabil Med.

